# Commitment to Sustainable Development Goals in marketing communications of ferry companies

**DOI:** 10.1371/journal.pone.0312767

**Published:** 2024-12-02

**Authors:** Natalia Wagner, Aleksandra Łapko, Ewa Hącia, Roma Strulak-Wójcikiewicz

**Affiliations:** Faculty of Economics and Transport Engineering, Maritime University of Szczecin, Szczecin, Poland; University of Central Punjab, PAKISTAN

## Abstract

Companies are not legally obliged to disclose their commitment to Sustainable Development Goals (SDGs). Despite this, many decide to build their competitive position on the basis of marketing communications about sustainability practices. This paper investigates the landscape of sustainability communications practices within ferry operators in the Baltic Sea Region. The authors have developed an index based on two sources of information: (1) an expert assessment of sustainability categories built on the basis of the SDGs, and (2) an assessment of marketing communications of ferry companies. The results of the study identified three distinct patterns of conduct. An analysis revealed that voyage safety and greenhouse gas emissions are the two categories which the ferry companies are most committed to. The results show that marketing communications on the realisation of social goals gives way to that on commitment to the realisation of environmental goals. Beyond insights into the ferry market, the study presents a valuable methodological tool for assessing sustainability communications across diverse industries.

## 1. Introduction

Making Sustainable Development Goals (SDGs) reality requires multiple actions taken by governments, institutions, and business entities. To successfully transform the world towards a sustainable future, the progress must be measured regularly and the results communicated clearly and transparently.

Assessing the contribution of businesses to achieving the SDGs is of utmost importance and, at the same time, complex for several reasons. Firstly, as a relatively new approach to business development, it is not yet based on a well-established methodology. Secondly, the ability to thoroughly analyse any activity undertaken by an enterprise is conditioned upon full transparency of processes and public disclosure of not only the financial standing, but also corporate sustainability records. Sustainability reporting is not mandatory for all companies, so a lot of businesses decide not to publish the data, which, by many, is considered sensitive. Thirdly, a lot of small and medium-sized enterprises do not keep relevant records, such as, e.g., data on CO_2_ emissions, which should be included in sustainability reports.

Much of the public’s attention has been focused recently on decarbonisation and sustainability initiatives taken by owners of container ships, bulk carriers and tankers. Those types of vessels have the highest fuel consumption globally and are thus major greenhouse gas emitters [1]. This is not to say, however, that owners of other types of ships, such as cruise ships or Ro-Pax ferries, do not engage in sustainability.

Research into sustainability disclosure, as published in the scientific literature, shows that relevant sustainability communication boosts a company’s image and financial results. Some businesses tie certain activities to selected SDGs [2]. Some, although not much, research into sustainability disclosure has been carried out for the shipping industry, with a major focus on container and cruise lines [3, 4].

The aim of this paper is to evaluate corporate sustainability communication practices applied by ferry operators in the Baltic Sea Region. The communications have been assessed for consistency of website contents and sustainability reports with sustainability criteria applicable to the ferry market and defined based on the Sustainable Development Goals (SDGs) and experts’ opinions.

The following research questions have been formulated:

Do ferry operators present themselves as sustainable brands through their marketing communications?

Which of the SDGs do ferry operators claim to contribute to?

The novelty of the research consists in the application of a research perspective, combining an experts survey and analysis of marketing messages, to the ferry transport sector in the Baltic Sea Region–a market where the realisation of the SDGs is still fairly unexplored. The authors have developed an index based on two sources of information: (1) an expert assessment of sustainability categories built on the basis of the SDGs, and (2) an assessment of marketing communications of ferry companies. Using the adopted research framework, twenty ferry companies operating in the Baltic Sea Region were analysed. Apart from interesting results concerning this particular market, a useful methodological tool for assessing the status of sustainability communications by business entities has been developed in the course of the study. The research framework can be applied to other shipping markets, and to other industries. The findings contribute to the corporate sustainability and SDGs-related literature, as well as research into management in the maritime transport.

The research object and the research method make the study unique, as compared to any prior research. Indeed, engagement of business entities, including those operating in the transport sector, in the realisation of the SDGs, and marketing communications as a tool for creating the image of a socially and environmentally responsible company, have been previously examined in scientific research [5, 6]. However, never before has the focus of such research been made on the ferry shipping industry or the specific character of its operations. The study object are ferry operators in the Baltic Sea Region, one of the major ferry shipping markets in the world [7]. It is characterised, among others, by the fact that the same ferry fleet is employed to handle passenger and cargo shipping [8]. Hence, the business strategies applied have to cover both market segments, and the marketing communications targeted to businesses as well as customers–passengers.

The study is organised as follows. The literature background in Section 2 includes an outline of challenges posed by the UN Sustainable Development Goals, results of recent research into the engagement of the shipping industry in the realisation of the SDGs, and an explanation of the role of marketing communications in this respect. In Section 3, the study object, i.e., ferry companies operating in the Baltic Sea Region, has been reviewed. The research method is described in Section 4, and Section 5 provides a discussion of the results. In Section 6, conclusions have been drawn, managerial implications proposed, and directions for future research indicated.

## 2. The literature background

### 2.1. Contribution to the realisation of the Sustainable Development Goals by the shipping industry

The Sustainable Development Goals (SDGs) and the 169 associated targets, adopted in the United Nations’ 2030 Agenda in 2015, are aimed to stimulate “action over the next 15 years in areas of critical importance for humanity and the planet” [9, p. 3]. The achievement thereof is conditioned upon overcoming challenges connected with the establishment of international policies, improving resource management, and a skilful integration of the synergy effect among the goals [10]. According to the Sustainable Development Goals Report 2023, a steady progress has been observed in the achievement of some of the SDGs. Nevertheless, progress towards some of the SDGs is unknown for lack of relevant data, whereas some of the SDGs are due to be delayed or the achievement thereof is in question, such as, e.g. SDG13 –Climate change [11].

The fact that the United Nations’ Agenda 2030 calls primarily upon the Member States to take action does not imply that enterprises should not get involved in the realisation of the SDGs or report progress in this respect. It must be emphasized here that the Agenda 2030 poses challenges also for companies, which should adjust their operations, strategies, and corporate sustainability reporting practices to the requirements of the SDGs [12]. Since the SDGs reporting is not mandatory, the information made public in this respect by enterprises in certain sectors is symbolic rather than substantial [13]. At the same time, in the face of competitive pressure, enterprises may tend to imitate one another’s sustainability reporting policies [14], including the practice of reporting on the SDG performance in their marketing communications [15, 16].

The major role of enterprises in the realisation of the Agenda 2030 and the inclusion of the SDGs in business strategies have been already recognized in the prior research literature [17–19]. Sustainability reporting with an indication of specific SDGs supported by the conducted business operations is becoming increasingly common [20]. The practice is applied especially by multinational corporations across a wide variety of industries [21]. Certain attempts to assess entire industry branches in terms of the SDGs prioritization, and to estimate the feasibility of realisation of the SDGs, have been made in a number of research studies [13, 22, 23]. In many of them, an analysis of the approach towards the realisation of the SDGs and the reporting on the same is broken down by the geographical region of operation and the type and nature of business organisation [24]. Various factors influencing the SDG adoption and reporting, such as, e.g., company size, financial performance, environmental risks, board size and structure, and regulatory context, are pointed out [25].

According to the principles of the Agenda 2030, achievement of all the SDGs is equally important to ensure a sustainable development of the world society. However, not all SDGs are equally relevant for a company [22, 26, 27] and companies enjoy the freedom of interpretation thereof and assigning them to the conducted business operations [28]. Initiatives have been taken to guide how to translate the SDGs into guidelines for sustainable business operation and contribute with company’s own activity to the realization of the SDGs [29, 30]. Moreover, organisations which develop sustainability reporting standards, such as, e.g., the GRI or the IIRC, have published guidance documents for inclusion of the SDGs into their framework [31, 32].

Viewed from another perspective, a discord has been observed between the SDGs realised for the common good and business goals of modern corporations aiming at increasing financial gains for the benefit of their shareholders and investors [18]. The discord is part of a broader discussion on the place and role of business in the modern world. An ideal solution would be to convert sustainable development challenges into business opportunities, which is difficult, but not impossible [33].

Enterprises which would assume social and environmental responsibility and provide sustainability reports in the past, tend to take a SDG-oriented approach to their current activity and marketing communications. Many similarities can be found between the concept of Corporate Social Responsibility (CSR) and the SDG-oriented approach. They are both built around the environmental, social, and economic aspects of business activity and modern societies. Nevertheless, the SDGs are defined in general terms and relate to entire societies or even the humanity, hence the incorporation thereof into actions which can be taken by business entities is an intricate task [15, 16, 18].

The shipping industry has already adopted the concepts of CSR and corporate sustainability as a basis for highlighting the contribution made to the achievement of the SDGs. Some of the SDGs, such as combating climate change, had been taken into consideration in the business strategies of some leading shipping companies even before the Agenda 2030 and the SDGs were established. The approach can be observed through an analysis of sustainability reports, websites, social media posts and other marketing communications by shipowners. It has been and continues to be embedded in the concepts of CSR and Corporate Sustainability, which, although have different roots, share the same vision of balancing economic responsibilities with social and environmental ones [34]. Nowadays, some shipowners attempt to incorporate them into the classification of the SDGs.

The CSR-related topics most commonly examined in research into the shipping industry include environmental issues (e.g. ballast water, oil spills, air pollution and emissions), diversity issues (e.g. gender unequal employment and/or payment issues), and business ethics (e.g. anti-corruption, anti-bribery) [35]. The type of CSR initiatives undertaken by shipping companies largely depends on the market segment they operate in and their business profile. For instance, shipowners operating on the cargo market focus on the anticipated customer expectations, and engage in solutions to environmental, occupational safety, rights of workers, and welfare issues [36]. Passenger shipping companies, on the other hand, apart from the activity in the area of environmental performance, which is common for all the shipping market segments, promote a wider inclusion of social and philanthropic aspects [37]. The sustainable approach onboard covers management practices and implementation of emerging technology solutions [38]. New technologies are applied in all areas on board–from digital navigation and data management through low-emission fuel solutions to waste management and education [39–41]. Nevertheless, the level of CSR maturity in the shipping industry is believed to be rather low compared to other industries [42], although research carried out in the Baltic Sea Region shows that shipping companies are slowly opening to the possible competitive advantage gains they may obtain through engaging in sustainable and responsible operations [43].

Realisation of the SDGs by the shipping industry is strongly endorsed by the International Maritime Organisation (IMO). As stated in the *IMO SDGs Strategy*, the IMO supports the Member States in the implementation of the 2030 Agenda as a whole, at the same time identifying the areas of most impact of its work, namely SDG14 (life below water), SDG13 (climate change), SDG9 (industry, innovation and infrastructure) and SDG5 (gender equality), and SDG17 (partnerships and resource mobilization) [44]. The measures implemented to this end by the IMO include primarily legislation; however, the IMO also acts as an educator and promotes as well as recommends activity to be taken by various entities of the Member States. The IMO has set a general direction for its Member States in their implementation of the 2030 Agenda, and has proposed a general interpretation of each of the SDGs with a focus on the feasibility of achievement of the same by the maritime sector and the maritime community. The possible activity in this respect is described in very general terms, leaving the administration authorities and business entities of the Member States free to take their own decisions.

Realisation of the SDGs in the maritime transport is not extensively discussed in the literature. Selected research papers are listed in Table 1. Kronfeld-Goharani, one of the first researchers dealing with the SDGs in the maritime transport, has highlighted corporate opportunities for strengthening the SDGs and contributing to a “sustainable blue growth” [45]. Di Vaio et al. [4] have undertaken to assess operations of four largest cruise and container ship owners and assign them to the realisation of individual SDGs. An analysis of sustainability reports for cruise shipping companies has revealed involvement in the realisation of all the SDGs. Container ship owners have not come out equally well in the analysis, as several SDGs have been found to remain unaddressed, namely SDG 6, 11, and 15. Nevertheless, in conclusion, leaders of the both market segments have been found to reveal a high level of commitment towards activity and reporting on sustainability issues, especially in the area of environmental impact. The analysed reports are fairly general on social goals and impact, with data presented descriptively rather than as facts and figures on the achieved outcomes. Efforts of all the shipowners under analysis towards clear communications aimed at sustainability disclosure have been assessed positively.

**Table 1:** Most relevant literature on SDG adoption and reporting in the shipping industry.

Authors	Reference	Main issue	Market segment	Method
**Xue and Lai**	[46]	A framework for understanding the concept of responsible shipping is proposed. The starting point for the analysis is SDG 12.	Shipping industry in general	Concept development
**Zhou et al.**	[47]	Analysis of reaction to and sentiment toward container shipping companies’ sustainability disclosure on social media.	Container shipping companies	Social media analysis (Twitter and Facebook)
**Di Vaio et al.**	[4]	Investigating the voluntary disclosure practices and environmental sustainability for achieving the SDGs in four leading cruise and container shipping companies.	Cruise and container shipping companies	Literature review and sustainability reports analysis
**Zhou et al.**	[3]	Analysing themes and patterns on how container shipping companies disclose sustainability.	Container shipping companies	Text mining applied to sustainability reports
**Wang et al.**	[6]	Examine contribution of maritime transport to implementation of SDGs. The concept of social entrepreneurship is also applied.	Container shipping liners and terminal operators	Sustainability reports analysis
**Di Vaio and Varriale**	[48]	Analysing the way in which the cruise industry could follow and achieve the sustainable goals, mostly SDG 11.	Cruise industry	Sustainability reports analysis
**Kronfeld-Goharani**	[45]	Understanding meaning of sustainability and the status of its implementation by corporations in the maritime economy. Based on example companies shows contribution of maritime industry to SDGs: 1, 3, 4, 7, 8, 9, 12, 13, 14, 16	Maritime industry in general	Analysis & discussion; Multi-case study

Another analysis of shipowners’ operations and assignment of SDGs has been performed by Wang et al [6]. The study sample was more extensive, as it covered 40 container shipping liners and 16 terminal operators. An interesting conclusion from the analysis is a breakdown of the SDG-related activities into three categories, aimed to help assess the SDG implementation status in the maritime economy. The key category are core responsibilities, including safe and healthy working environment (from the area of SDG 8), development of green technologies and transport infrastructure (SDG 9), responsible waste management and ship recycling (SDG 12), and ballast water management and coastal ecosystem protection (SDG 14). The other two categories are considered additional and cover extended responsibilities. The study discussed herein is also based on detailed categories of activities rather than on broadly defined SDGs.

Prior research work into the shipping industry and the SDGs examines sustainability reports and social media communications without a focus on particular SDGs but rather referring to a general message shared by all the SDGs jointly. Within this approach, Zhou et al. [3] have proposed three major categories of activities which container shipping companies should report on, namely employee training and management, sustainable business management, and sustainable ship operation. They are further broken down into more detailed subcategories.

A new research approach–applied, inter alia, to the shipping industry–is an analysis of sustainability disclosure in social media. According to a ranking of SDGs as perceived by stakeholders in social media (Twitter and Facebook), developed by Zhou et al. [47], the most important SDGs in the opinion of stakeholders are SDG 6, 14, 5, 8, and 11.

In a great majority of research studies, the tasks of the maritime transport sector in the area of the SDG implementation are analysed across all or nearly all the SDGs. The approach is applied in studies focusing on shipowners only as well as in those which investigate the entire blue economy including, inter alia, oil and gas industry, marine renewable energy production, aquaculture, port activities, sea tourism, or shipbuilding [49, 50]. The same approach has been taken in this study. It is worth noting here, however, that research is in progress investigating the commitment into and outcomes of broadly defined economic and political activity in the maritime sector with reference to one SDG only, most commonly SDG 14 –life below water [51, 52], which can be classified as a goal in the area of natural environment. Although it seems to be a justified approach, Xue and Lai [46] reiterate that the social and economic aspects of the maritime transport operations must not be ignored, and that the greatest challenge faced on the way towards the sustainable development of maritime transport is SDG 12 (sustainable production and consumption), which corresponds to the current goals of the economic development directly influencing the quantities of cargo carried by sea.

### 2.2. The role of marketing communications in building the social awareness of sustainable development

Although shipowners are becoming increasingly committed to the implementation of an SDG-oriented strategy and pay more consideration to activities in the area of CSR, their efforts may go unnoticed in the public awareness. According to Zatwarnicka-Madura et al., appropriate communication to the stakeholders is key to being able to use the leverage of efforts in this respect and translate them into measureable business gains [53]. Appropriate marketing communications and informing about the company’s commitment into the implementation of a sustainable development policy can boost the company’s image and its competitive edge [54]. Such information can be meaningful to stakeholders, have an impact on their purchasing decisions, reduce uncertainty, validate the company’s decisions and add quality to the services rendered [55]. Moreover, proper marketing communications build consumer awareness, leading to increased expectations concerning the practices applied by companies. Thus, they can stimulate the realisation of responsible practices and contribute to the achievement of sustainable development goals [55]. Therefore, enterprises should go beyond mandatory reporting obligations and ensure that information about their sustainability performance reaches the largest possible audience [56, 57].

The use of the Internet and Internet-based tools is of key importance here. The Internet has as many as 5.18 billion users worldwide, which amounts to 64.6% of the global population [58]. It is the major source of commercial information and has a significant impact on consumer decisions [59, 60]. Convenience of Internet-based communication lies in the fact that it helps curb costs and provides immediate access to contents as well as an opportunity to get consumer feedback [61]. One of the basic web-based tools are websites, considered both by companies and stakeholders as a major channel of communication and an enterprise showcase [62]. Depending on complexity, they may offer various functionalities, such as, e.g., contact data, location, offering of services, educational materials, booking and payment facilities [63]. They may also provide information on the CSR performance and the implemented SDGs [64]. A company website integrated with the company’s social media profiles becomes an even more powerful, wide-range tool of content distribution [65]. Information on SDG performance posted on a company website or in the social media may serve an educational role to consumers browsing for information before using the company’s services [66]. Marketing communications help consumers make informed choices and add to the popular understanding of the necessity to achieve the SDGs [67]. As highlighted by Redman and Wiek, an informed society is a basis for transformations towards sustainability [68]. Thus, realisation of the SDGs and communicating the same to the stakeholders can contribute to the building of consumer awareness, popularising the idea, and driving a positive change.

## 3. An overview of passenger ferry shipping in the Baltic Sea

**Fig 1:**
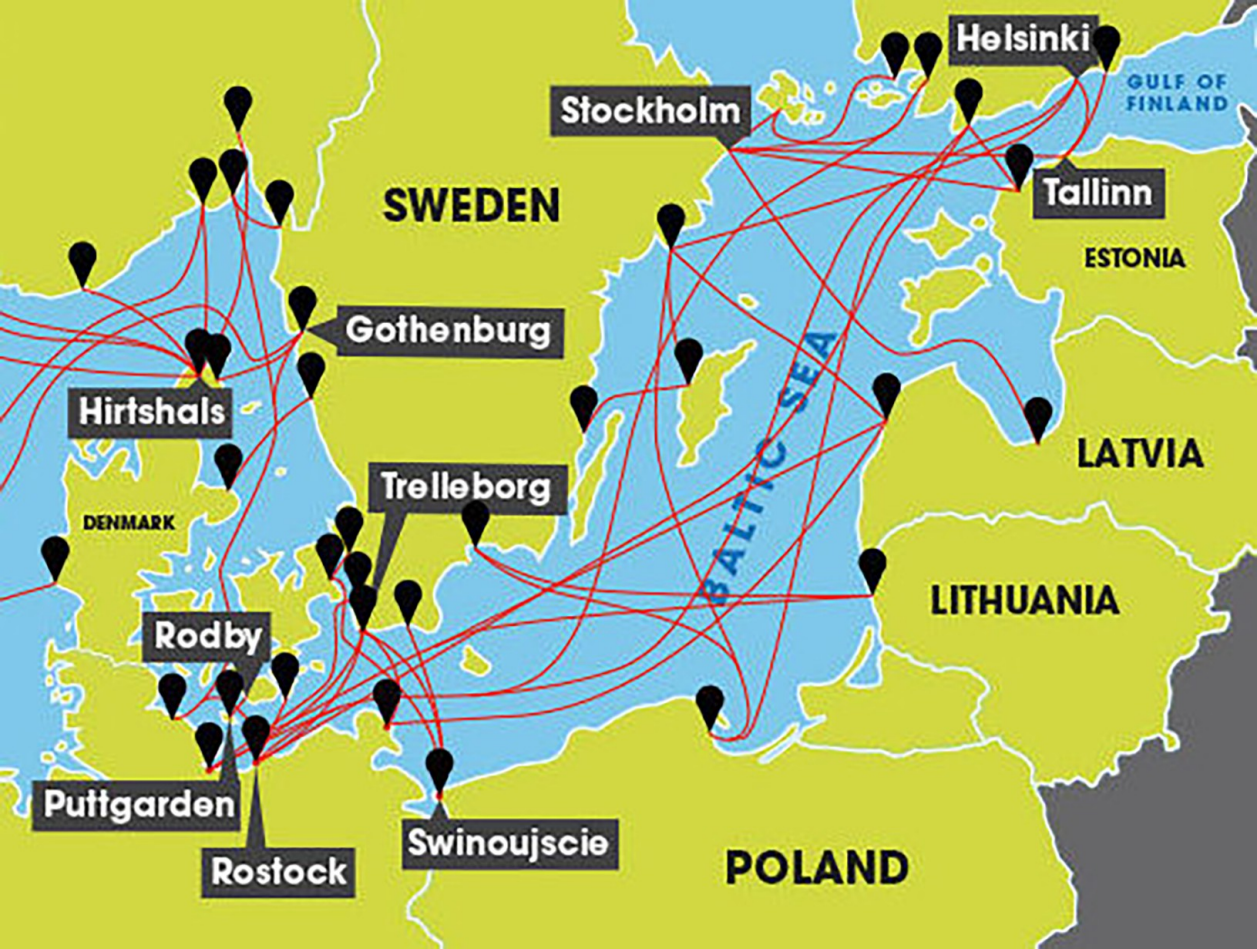
Main ferry connections between the Baltic countries (Sweden, Finland, Russia, Estonia, Latvia, Lithuania, Poland, Germany, and Denmark). Source: Reprinted from [71] under a CC BY license, with permission from Freightlink Solutions Ltd, original copyright 2023.

The Baltic Sea is one of the largest brackish water bodies in the world, covering an area of 420 000 km^2^. It is a semi-enclosed shallow sea with an average depth of 60 m, where one third of the area is less than 30 m deep [69]. It is one of the busiest passenger shipping areas in the world [70]. The Baltic countries, including Sweden, Finland, Russia, Estonia, Latvia, Lithuania, Poland, Germany, and Denmark, have well-established ferry connections (Fig 1).

There are over 50 passenger ferry terminals and harbours in the Baltic Sea which handle passenger shipping on a regular basis. Some harbours feature one ferry terminal, others have more than one (e.g. Tallinn, Helsinki, Turku, and Stockholm). The offering of ferry voyages in the Baltic Sea is very diverse, from 30-minute passages on small, 50-person ferry boats to overnight voyages onboard ferries which can accommodate up to 3,000 passengers. Most of the voyages in the northern Baltic Sea are overnight, the southern Baltic Sea is criss-crossed with short-distance ferry connections e.g., from Poland and Germany to Denmark and Sweden. There are at least 20 ferry operators in the Baltic Sea offering passenger and car ferry services. Uniqueness of the area lies in the fact that on some routes, the smallest ferry operators can successfully compete with giants. Many of the Baltic countries feature an insular coast, and passenger ferry connections are used to commute to work or shop for food and household supplies [72].

A list of main international ferry routes in the Baltic Sea Region and major ferry operators is shown in Table 2. The list includes ferry routes which start and finish in the Baltic Sea, and so is exclusive of ferry routes to Norway or Denmark, as well as of domestic ferry routes (e.g. in Poland, Germany, the Åland Islands, etc.).

**Table 2:** Ferry routes in the Baltic Sea Region and main ferry operators.

no.	Ferries from	Ferry Route	Ferry Operator(s)
**1**	Finland	Helsinki to Lübeck/Travemünde (Germany)	Finnlines
		Helsinki to Tallinn (Estonia)	Tallink-Silja Line, Viking Line, Eckerö Line
		Helsinki to Stockholm (Sweden) via Åland	Tallink-Silja Line, Viking Line
		Helsinki to Åland	Tallink-Silja Line, Viking Line
		Vaasa to Umeå (Sweden)	Wasaline
		Lappeenranta to Vyborg (Russia) via Saimaa Canal	Saimaa Travel
		Turku to Stockholm (Sweden) via Åland	Tallink-Silja Line, Viking Line, Finnlines
		Turku to Åland	Tallink-Silja Line, Viking Line, Finnlines
		Hanko to Nynäshamn (Sweden)	Stena Line
**2**	Estonia	Tallinn to Stockholm (Sweden)	Tallink Silja, Viking Line
		Tallinn to Stockholm (Sweden) via Åland	Tallink Silja, Viking Line
		Tallinn to Helsinki (Finland)	Tallink-Silja Line, Viking Line, Eckerö Line
		Paldiski to Kapellskär (Sweden)	DFDS
		Rohuküla to Hiiumaa	TS Laevad OÜ (praamid.ee)
		Virtsu to Muhu+Saaremaa	TS Laevad OÜ (praamid.ee)
		Leppneeme to Kelnase	Tuule Liinid
		Tallinn to Aegna	Kihnu Veeteed
		Laaksaare to Piirissaare	Kihnu Veeteed
		Munalaid to Kihnu	Kihnu Veeteed
		Munalaid to Manilaid	Kihnu Veeteed
		Saaremaa (Triigi) to Hiiumaa (Sõru)	Kihnu Veeteed
		Rohuküla to Vormsi (Sviby)	Kihnu Veeteed
**3**	Latvia	Ventspils to Nynäshamn (Sweden)	Stena Line
		Liepāja to Lübeck/Travemünde (Germany)	Stena Line
**4**	Lithuania	Klaipėda to Kiel (Germany)	DFDS Seaways
		Klaipėda to Karlshamn (Sweden)	DFDS Seaways
		Klaipėda to Trelleborg (Sweden)	TT-Line
		Klaipėda to Rostock (Germany)	TT-Line
		Klaipėda to Lübeck/Travemünde (Germany)	TT-Line
**5**	Poland	Gdynia to Bałtyjsk (Kaliningrad Oblast, Russia)	Żegluga Gdańska
		Świnoujście to Ystad (Sweden)	Unity Line, Polferries
		Gdańsk to Nynäshamn (Sweden)	Polferries
		Świnoujście to Trelleborg (Sweden)	TT-Line
		Gdynia to Karlskrona (Sweden)	Stena Line
		Kołobrzeg to Bornholm (Denmark)	Kołobrzeska Żegluga Pasażerska
**6**	Germany	Lübeck/Travemünde to Helsinki (Finland)	Finnlines
		Kiel to Klaipėda (Lithuania)	DFDS Seaways
		Lübeck/Travemünde to Liepāja (Latvia)	Stena Line
		Rostock to Klaipėda (Lithuania)	TT-Line
		Rostock to Trelleborg (Sweden)	Stena Line, TT-Line
		Rostock to Gedser (Denmark)	Scandlines
		Sassnitz to Ystad	FRS Baltic—FRS Königslinjen
		Puttgarden to Rødby (Denmark)	Scandlines
		Sassnitz to Bornholm (Denmark)	Bornholmslinjen
		Kiel to Gothenburg	Stena Line
		Lübeck/Travemünde to Malmö (Sweden)	Finnlines
		Lübeck/Travemünde to Trelleborg (Sweden)	TT-Line
		Kiel to Gothenburg	Stena Line
**7**	Denmark	Bornholm to Kołobrzeg (Poland)	Kołobrzeska Żegluga Pasażerska
		Bornholm to Ystad (Sweden)	Bornholmslinjen
		Køge to Bornholm	Bornholmslinjen
		Bornholm to Sassnitz (Germany)	Bornholmslinjen
		Copenhagen (Denmark) to Świnoujście (Poland)	Polferries
		Copenhagen to Oslo	DFDS Seaways
		Frederikshavn to Oslo	DFDS Seaways
**8**	Sweden	Stockholm to Turku (Finland) via Åland	Tallink-Silja Line, Viking Line, Finnlines
		Stockholm to Helsinki (Finland) via Åland	Tallink-Silja Line, Viking Line
		Nynäshamn to Hanko (Finland)	Stena Line
		Stockholm (Grisslehamn) to Eckerö (Åland)	Eckerölinjen
		Kapellskär to Paldiski (Estonia)	DFDS, Tallink-Silja Line
		Nynäshamn to Ventspils (Latvia)	Stena Line
		Karlshamn to Klaipėda (Lithuania)	DFDS Seaways
		Trelleborg to Klaipėda (Lithuania)	TT-Line
		Ystad to Świnoujście (Poland)	Unity Line, Polferries
		Nynäshamn to Gdańsk (Poland)	Polferries
		Trelleborg to Świnoujście (Poland)	TT-Line
		Karlskrona to Gdynia (Poland)	Stena Line
		Trelleborg to Rostock (Germany)	Stena Line
		Malmö to Lübeck/Travemünde (Germany)	Finnlines
		Trelleborg to Lübeck/Travemünde (Germany)	TT-Line
		Ystad to Bornholm (Denmark)	Bornholmslinjen
		Oskarshamn to Visby	Destination Gotland
		Nynäshamn to Visby	Destination Gotland
		Gothenburg to Kiel	Stena Line
	Russia	Cruises suspended	

Source: own elaboration on the basis of [73]

Ferries of the Ro-Pax type, which have both passenger spaces and holds for vehicles and other ro-ro cargo, are operated on most of the routes. Ro-Pax ferry boats feature passenger cabins, seats on the main deck, restaurants, shops and entertainment spaces. Vehicles are driven into the holds and parked there for the voyage. At the port of destination, vehicles are driven out of the ferry boat [72].

The legal regulations applicable in the Baltic Sea Region are stricter than those in other parts of the world, due to its hydrological structure and a marine ecosystem which requires special protection [74]. The limits of SOx and NOx emissions which apply in the Baltic Sea, as well as in the North Sea, are lower than anywhere else in the world. Therefore, shipping companies operating in the area invest in innovative and environmentally friendly ferry boats powered by alternative fuels. Ferry operators in the Baltic Sea Region should base their business strategies on environmental activity and gain competitive edge through offering high service quality [72].

## 4. Research methods

**Fig 2:**
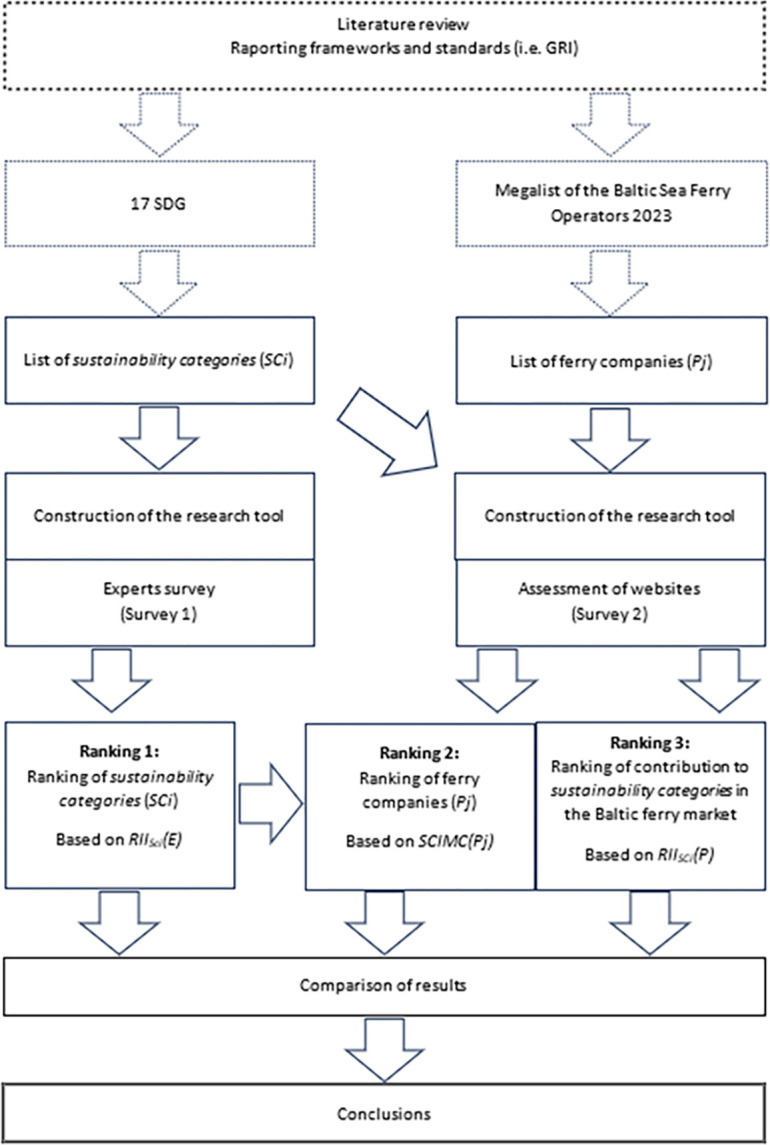
Research procedure.

A diagram of the research procedure comprising all the research phases is shown in Fig 2.

In the first step of the research, ten areas, referring to the UN Sustainable Development Goals and the 169 tasks, which may be addressed in the marketing communications of ferry companies, have been selected. In their choice, the authors supported themselves with the reporting frameworks and standards (i.e., GRI), and the academic literature. The selected areas have been named *sustainability categories* (*SCi*). Listed in Table 3, they are assigned to selected SDGs. For information purposes and better visualisation, the graphical icons developed by the UN for particular sustainable development goals have been used.

**Table 3:** List of *sustainability categories* (*SCi*).

Symbol [SCi]	*Sustainability category* name	Assignment to SDGs
**SC1**	Air pollution emissions	SDG 11, SDG 13, SDG 14
**SC2**	Greenhouse gas emissions	SDG 11, SDG 13, SDG 14
**SC3**	Energy and water consumption	SDG 6, SDG 7
**SC4**	Waste management	SDG 12, SDG 14
**SC5**	Food waste mitigation	SDG 2, SDG 12, SDG 14
**SC6**	Social involvement	SDG 4, SDG 5, SDG 9
**SC7**	Application of a code of ethics	SDG 5, SDG 10
**SC8**	Cooperation with local partners and suppliers	SDG 8, SDG 12, SDG 17
**SC9**	Voyage safety	SDG 3, SDG 9
**SC10**	Support provided to passengers with special needs (e.g., disabilities)	SDG 3, SDG 9, SDG 10

**Fig 3:**
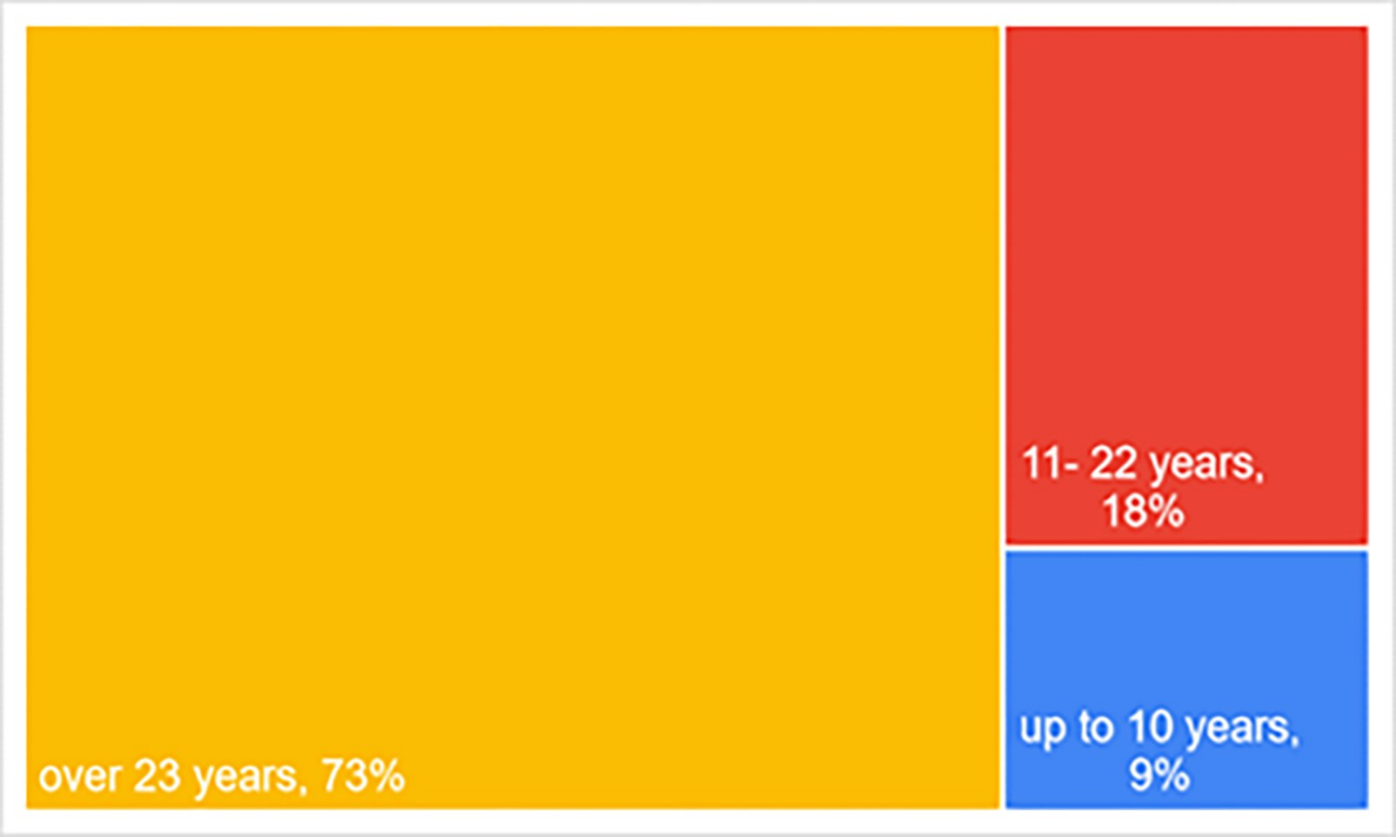
Breakdown of experts participating in the survey by years of experience in the maritime transport/tourism [%].

The experts survey (Survey 1) was conducted at the end of March and the beginning of April 2023 using the Computer Assisted Web Interview (CAWI), which is a type questionnaire survey. The questionnaire was developed in Google Forms and mailed to 11 experts selected as a non-random sample. The selection criterion was an at least several-year professional experience in maritime transport or tourism (with a special focus on sea tourism). A great majority of experts in the study sample have more than 23 years of experience (Fig 3). They conduct scientific research and draw up expert’s opinions for businesses. Written informed consent was obtained from all experts involved in the study.

The experts survey was aimed to develop a ranking of importance of the *sustainability categories* (Ranking 1) listed in Table 3. The first question asked whether ferry operators should refer to the UN Sustainable Development Goals (17 SDGs) in marketing communications. Further questions asked whether websites of ferry operators should contain information on: air pollution emissions, greenhouse gas emissions, energy and water consumption, waste management, food waste mitigation, social involvement, application of a code of ethics, cooperation with local partners and suppliers, voyage safety, and support provided to passengers with special needs (e.g., disabilities); i.e., individual *sustainability categories*. Experts responded using a 5-point Likert scale (5 –I definitely agree, 4 –I agree, 3 –neutral, 2 –I disagree, 1 –I definitely disagree).

The ranking of *sustainability categories*, i.e., topics which, according to the experts, should be addressed on the website of a ferry operator, has been developed on the basis of the relative importance index (*RII*) [75], modified as follows:

$${RII}_{SCi}(E)=\frac{5n_{5}+4n_{4}+3n_{3}+2n_{2}+1n_{1}}{5(n_{5}+n_{4}+n_{3}+n_{2}+n_{1})},$$ (1)

where *n*_*1*_, *n*_*2*_, *n*_*3*_, *n*_*4*_ and *n*_*5*_ the number of respondents (experts) who selected from 1 to 5.

According to the value of *RII*_*SCi*_*(E)*, each SCi was assigned a weight, where the top position in the ranking had a weight of 9, and weights of the following positions descended down to the bottom position with a weight of 1. Two SCis whose values of *RII*_*SCi*_*(E)* were equal were assigned the same weights.

Survey 2 consisted in an assessment of the SDG-related marketing communications on websites and in reports of ferry operators in the Baltic Sea Region.

The ferry companies under analysis have been selected on the basis of the *Megalist of Baltic Sea Ferry Operators 2023* [76]. These are all operators serving the Baltic Sea market at that time. They are listed in Table 4.

**Table 4:** Ferry companies operating in the Baltic Sea Region (*Pj*).

Symbol [*Pj*]	Company name	Symbol [*Pj*]	Company name
**P1**	Finnlines	P11	Bornholmslinjen
**P2**	Viking Line	P12	Eckerölinjen
**P3**	Tallink	P13	Destination Gotland
**P4**	Eckerö Line	P14	Scandlines
**P5**	StenaLine	P15	Żegluga Gdańska
**P6**	DFDS	P16	Kołobrzeska Żegluga Pasażerska Sp. z o.o.
**P7**	TT-Line	P17	TS Laevad OÜ (praamid.ee)
**P8**	Wasaline	P18	Kihnu Veeteed
**P9**	Unity Line	P19	Tuule Liinid
**P10**	Polferries	P20	Saimaa Travel

Marketing communications on websites and in reports of the ferry operators under analysis have been assessed for inclusion of information referring to the *sustainability categories* listed in Table 3. A 6-point scale has been used, as follows: 0 –No information, 1 –The topic is mentioned, 2 –The topic is mentioned and an extensive explanation included, 3 –One example of action is provided, 4 –More than one example of action is provided, 5 –Information is provided in a separate tab/file/document.

Using the weights from Ranking 1, obtained in Survey 1, in combination with the assessment of marketing communications obtained in Survey 2, the level of involvement of ferry companies and the entire ferry shipping market in the performance of actions assigned to the sustainability categories has been assessed.

Taking into consideration the weights of individual *sustainability categories* (based on Ranking 1), total weighted marks have been calculated, which can be construed as representing commitment into the realisation of selected SDGs as mentioned in the marketing communications by the enterprises. For this purpose, an original concept of the *SDGs Commitment Index in Marketing Communication* (*SCIMC*) index has been developed, calculated from the following formula:

$$SCIMC(Pj)=\frac{\sum_{i=1}^{10}o_{SCi}(Pj)∙w(SCi)}{\sum_{i=1}^{10}w(SCi)},$$ (2)

where: *o*_*SCi*_*(Pj)*–mark granted to the company website for a particular SCi, *w(SCi)*–weight assigned to that *SCi*.

The *SCIMC(P*_*j*_*)* index can have a value of 0 to 5. The higher the index value, the higher the commitment to the realisation of the SDGs represented in marketing communications. The *SCIMC(P*_*j*_*)* index has been used to establish a ranking of enterprises (Ranking 2). Depending on the index value, the level of commitment into the realisation of the SDGs represented in the marketing communications, can be described as: high (at least 4.00), medium (at least 3.00 but less than 4.00), low (at least 2.00 but less than 3.00), and very low (less than 2.00). Calculated for a single enterprise, the *SCIMC(P*_*j*_*)* index shows its commitment to the SDGs in the marketing communications.

Survey 2 consisted in an analysis of the selected enterprises and another one of the entire Baltic ferry market as a whole. Ranking 3 developed on the basis of Survey 2 showed contribution to sustainability categories in the Baltic ferry market. For this purpose, i.e., in order to establish a ranking of *sustainability categories* by their representation on websites of the 20 ferry companies under analysis jointly, the relative importance index (RII) [75] was used again, this time modified as follows:

$${RII}_{SCi}(P)=\frac{5o_{5}+4o_{4}+3o_{3}+2o_{2}+1o_{1}}{5(o_{5}+o_{4}+o_{3}+o_{2}+o_{1}+o_{0})},$$ (3)

where o_0_, o_1_, o_2_, o_3_, o_4_ and o_5_ is the number of companies granted a mark from 0 to 5.

## 5. Results and discussion

The first question in the experts questionnaire survey (Survey 1) asked about the validity of creating marketing communications referring to the 17 SDGs. A great majority of experts acknowledged that ferry operators should refer to the SDGs in their marketing communications, only one expert acknowledged the opposite. Based on the responses provided to other questions, a ranking of *sustainability categories* has been created (Table 5 - Ranking 1). The topics of key importance, which, according to the experts’ opinion, should be addressed on websites of ferry companies, are voyage safety (SC9) and support for passengers with special needs, including disabilities (SC10). Cooperation with local partners and suppliers (SC8) and food waste mitigation (SC5) ranked at the bottom. The ranking is shown in Table 5, with weights assigned to each SCi. The experts questionnaire survey was validated with internal consistency by calculating Cronbach’s alpha. The result equals 0.941 which is above the threshold criterion of 0.7 and means that the data can be regarded as highly acceptable [77].

**Table 5:** Ranking of *sustainability categories* (Ranking 1).

Rank	Symbol [*SCi*]	Description	*RII*_*SCi*_ *(E)*	Weight [*w(SCi)*]
**1**	SC9	Voyage safety	0.91	9
**2**	SC10	Support provided to passengers with special needs (e.g., disabilities)	0.89	8
**3**	SC4	Waste management	0.75	7
**3**	SC7	Application of a code of ethics	0.75	7
**4**	SC1	Air pollution emissions	0.73	6
**5**	SC2	Greenhouse gas emissions	0.71	5
**6**	SC6	Social involvement	0.69	4
**7**	SC3	Energy and water consumption	0.67	3
**8**	SC8	Cooperation with local partners and suppliers	0.65	2
**9**	SC5	Food waste mitigation	0.64	1

An analysis of the contents of websites (Survey 2) has revealed a varied approach to communications on commitment to the SDGs. Although the companies operate in the same area and many of them compete with one another, they do not follow a single standard of marketing communications concerning sustainability. Only six out of twenty companies under analysis publish a sustainability/ESG report, and only five of them declare commitment to specific SDGs (Table 6). Other companies, although do not publish sustainability reports or refer to any specific SDGs relevant to their operations, communicate their priorities and activity in this area. Therefore, in order to assess sustainability communications and the companies’ commitment to the realisation of the SDGs, it is recommended to extend the research by evaluating the sustainability criteria related to specific SDGs.

**Table 6:** Publication of a sustainability report by the ferry companies under analysis.

Symbol [*Pj*]	Company name	Publishes Sustainability/ESG/Integrated Report	The SDG referred to
**P1**	Finnlines	√	8, 9, 12, 13, 14
**P2**	Viking Line	√	3, 7, 12, 14
**P3**	Tallink	√	3, 6, 7, 11, 12, 13, 14, 15
**P4**	Eckerö Line	-	-
**P5**	StenaLine	√	3, 5, 7, 10, 12, 14
**P6**	DFDS	√	-
**P7**	TT-Line	-	-
**P8**	Wasaline	-	-
**P9**	Unity Line	-	-
**P10**	Polferries	-	-
**P11**	Bornholmslinjen	-	-
**P12**	Eckerölinjen	-	-
**P13**	Destination Gotland	√	5, 9, 13, 14
**P14**	Scandlines	-	-
**P15**	Żegluga Gdańska	-	-
**P16**	Kołobrzeska Żegluga Pasażerska Sp. z o.o.	-	-
**P17**	TS Laevad OÜ (praamid.ee)	-	-
**P18**	Kihnu Veeteed	-	-
**P19**	Tuule Liinid	-	-
**P20**	Saimaa Travel	-	-

**Fig 4:**
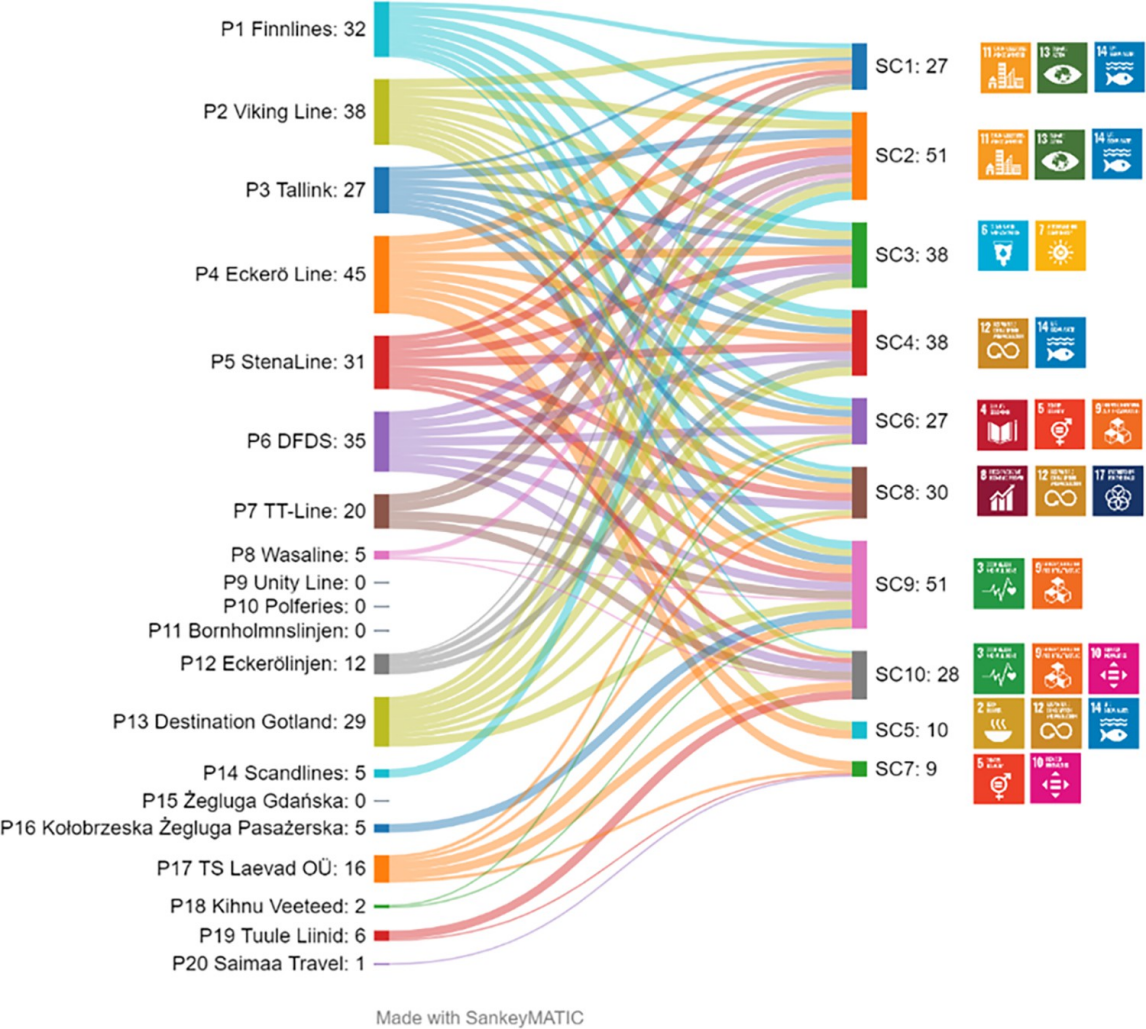
Strength of connections between marketing communications of the ferry operators under analysis and the sustainability categories/ SDGs.

The Sankey diagram in Fig 4 demonstrates which sustainability categories are referred to most in marketing communications of the enterprises under analysis. It also shows the total of points obtained by each enterprise and the sustainability categories to which the points are assigned. Eckerö Line scored the highest in all the examined categories, save for SC10. Some of the enterprises under analysis scored zero in all the categories. The Sankey diagram also shows which categories are most often referred to in the marketing communications of all the companies jointly. SC2 and SC9 rank at the top, which means that sustainability communications by operators in the Baltic ferry market covers primarily greenhouse gas emissions and voyage safety. Hence, it can be concluded that sustainability communications in the Baltic ferry market is built around SDG 3, 9, 11, 13, and 14.

The information gathered was used to calculate the *SCIMC(P*_*j*_*)* index. Table 7 shows a ranking of the ferry companies under analysis, by the *SCIMC(P*_*j*_*)* index value–Ranking 2.

**Table 7:** Ranking of ferry companies (Ranking 2).

Rank	Symbol [*Pj*]	Company name	*SCIMC(Pj)*	Rank	Symbol [*Pj*]	Company name	*SCIMC(Pj)*
**1**	P4	Eckerö Line	4.23	11	P19	Tuule Liinid	0.90
**2**	P6	DFDS	3.65	12	P16	Kołobrzeska Żegluga Pasażerska Sp. z o.o.	0.87
**3**	P2	Viking Line	3.58	13	P8	Wasaline	0.62
**4**	P1	Finnlines	3.31	14	P14	Scandlines	0.48
**5**	P5	StenaLine	3.31	15	P18	Kihnu Veeteed	0.25
**6**	P13	Destination Gotland	3.00	16	P20	Saimaa Travel	0.13
**7**	P3	Tallink	2.77	17	P9	Unity Line	0.00
**8**	P7	TT-Line	2.69	18	P10	Polferries	0.00
**9**	P17	TS Laevad OÜ (praamid.ee)	2.13	19	P11	Bornholmnslinjen	0.00
**10**	P12	Eckerölinjen	1.17	20	P15	Żegluga Gdańska	0.00

Here, the maximum constituent scores have been obtained for SC1 –SC5. No references have been found to the application of a code of ethics (SC7). The application of a code of ethics receives the least coverage on the websites of the enterprises under analysis, despite the fact that according to the surveyed experts it is a topic of high importance (the fourth position in the ranking of experts’ opinions). Equally rare is information on food waste mitigation (SC5); however, this aspect of realisation of the SDGs has been indicated by the experts as the least important in marketing communications. The enterprises in positions three to six have obtained similar values of the *SCIMC(Pj)* index (within a range of 3.00 do 3.58). The maximum constituent scores for the websites of those enterprises have been obtained for information on greenhouse gas emissions (SC2), energy and water consumption (SC3), waste management (SC4), and voyage safety (SC9). As many as eleven enterprises communicate the realisation of the SDGs at a very low level of the *SCIMC(Pj)* index of less than 2.0. Four enterprises have scored zero, i.e., they do not post any information on the realisation of the SDGs.

**Fig 5:**
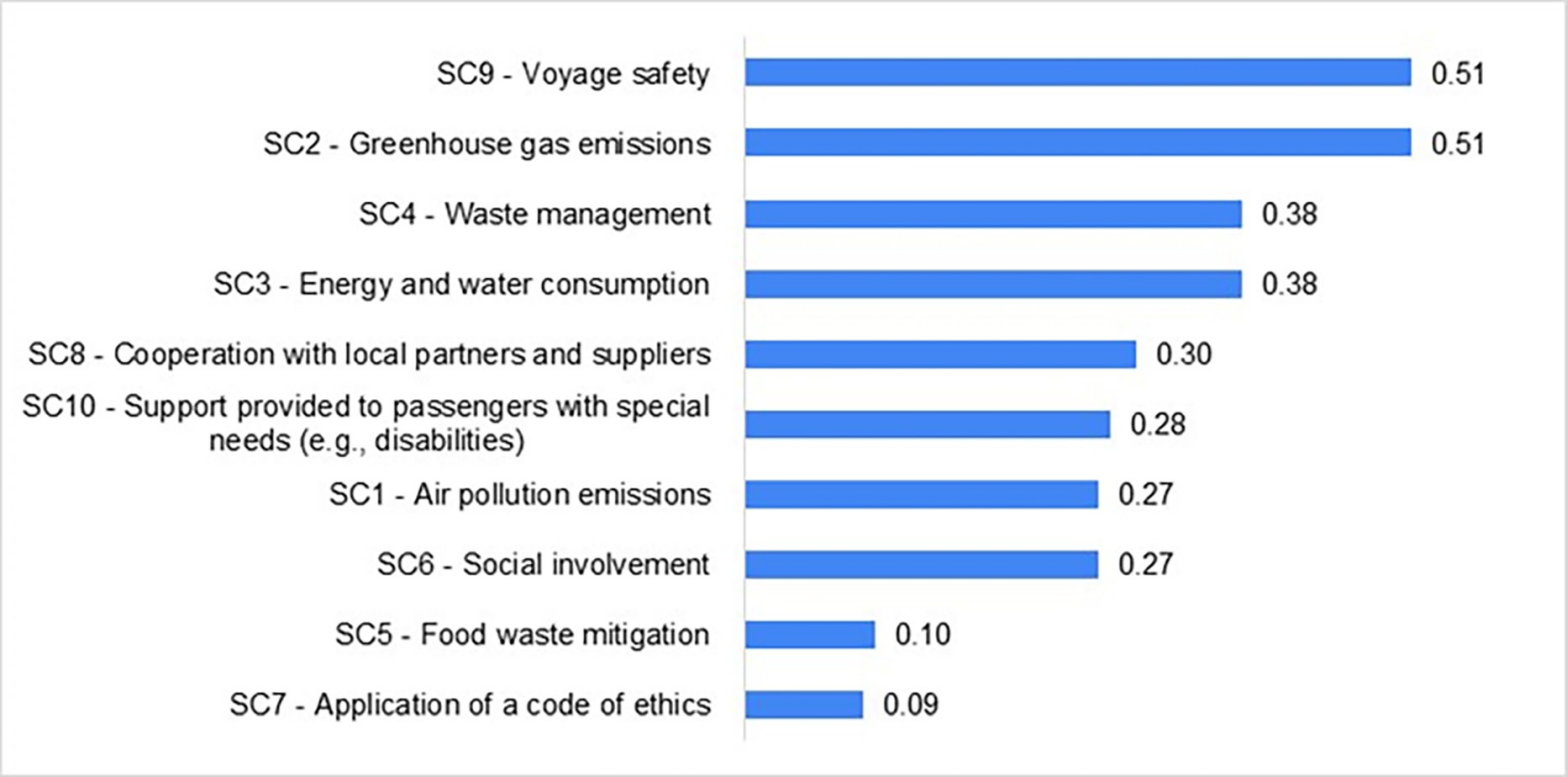
Ranking of contribution to sustainability categories by the Baltic ferry market (Ranking 3).

Subsequently, calculations have been made for the entire Baltic ferry market. Ranking 3 of contribution to sustainability categories by the Baltic ferry market, by the *RII*_*SCi*_*(P)* index, is shown in Fig 5. A comparison of Ranking 3 with the results of the experts survey about the validity of creating marketing communications referring to the 17 SDGs (Table 5) shows that part of the information considered by the experts to be of key importance is never provided on websites of many of the enterprises under analysis. The correlation between the weight assigned on the basis of the experts’ opinion and the presence of the information on the websites is not observed in all the cases.

Voyage safety (SC9) ranks at the top in both rankings. According to the information on the websites, the enterprises attach a lot of importance to greenhouse gas emissions (SC2), a category which has been considered by the experts as of low importance and ranked sixth. According to the experts, information on food waste mitigation (SC5) is of least importance; equally low importance is attached to it by the companies under analysis in their marketing communications. Support provided to passengers with special needs (SD10), highly regarded by the experts, has ranked at the bottom half of the ranking of websites. Waste management (SC4) has ranked third in both rankings.

SC2 –greenhouse gas emissions–deserves special attention in view of the introduction of new legal regulations. Ro-Pax ferries are characterised by a relatively lower fuel consumption; e.g., compared to container ships, by 6.7% on international routes [1]. However, despite the fact that emissions in the Baltic Sea are lower than in other shipping markets, ferry lines need to adjust to new circumstances of operation based on the cornerstones of sustainable development. Within the European Union, the principles of operation are imposed not only under the IMO regulations, but also under the EU legal framework, including the recently introduced regulations aimed at combating climate change, such as the ‘Fit for 55’ package and the EU taxonomy. The time horizon for the introduction of necessary changes is short-term, taking into consideration the high investments which have to be made by shipowners as well as sea harbours. One example of the changes to be implemented is the requirement that passenger ferries and containerships at berth in an EU port be connected to an onshore power supply (OPS), which is to come into force as of January 1, 2030. Exemptions from the requirement shall apply to vessels laying at berth for under two hours and emergencies [78].

## 6. Conclusions

Currently, ferry operators are not legally obliged to report on the manners or scope of realisation of the sustainable development goals. Enterprises may decide to do so at their own discretion; however, many decide not to, considering the relevant information and data as sensitive. Meanwhile, research results have shown that making information on the ferry operator’s commitment to the realisation of the SDGs available to the public is a meaningful part of marketing communications. According to experts’ opinion, potential customers may attach importance to such information and rely on it when choosing a ferry operator. The following categories of information have been considered of special importance by the experts: voyage safety, support provided to passengers with special needs (e.g., disabilities), waste management, and application of a code of ethics. A fact worth noting here is that the two categories which have been assigned the highest weights are directly related to social goals. A study of the literature has shown that activity aimed at realising social goals receives the least coverage in marketing communications of enterprises operating in the maritime transport. Most of them focus on tasks related to environmental protection.

This paper examines marketing communications of ferry operators related to the realisation of sustainable development goals. Considering the specific character of their business operations, ferry companies need to address not only business clients (transport companies, carriers, tourist companies), but also individual customers (tourists and other passengers).

The study has provided answers to the posed research questions. The first research question asked whether ferry shipowners present themselves as sustainable brands through marketing communications **(RQ1)**. The results of the study show that sustainability marketing communications by ferry shipowners is varied and no single standard has been developed in this regard. Three patterns of conduct have been identified. One is based on publishing contents on corporate sustainability and direct references to the SDGs. Out of twenty enterprises under analysis, five have been found to follow this pattern (Finnlines, Viking Line, Tallink, StenaLine, and Destination Gotland). What is interesting, the leader in the ranking of enterprises by the SCIMP(Pi) index does not fall into this group. The SCIMP (Pj) index values calculated for these five enterprises have been defined as medium.

Another pattern consists in informing about the activity aimed at contribution to the sustainable development goals but not using the SDG-related terminology. This pattern is followed, inter alia, by Eckerö Line, which ranked at the top in the ferry shipowners ranking as the only ferry operator whose SCIMP index value is within the ‘high’ range. What is interesting, the company operates only two ferry boats on the Helsinki–Tallin route. Two other ferry companies, Tallink-Silja Line and Viking Line, operate on the same route using a larger number of ferry boats and communicating on sustainability issues on their websites. Therefore, all the ferry operators competing on the same route attach a lot of attention to sustainability communications. It may suggest that communications on the realisation of the SDGs serve as a competitive edge. The latter has been confirmed in previous research described in the literature, whose results suggest that the increasing social awareness of the necessity to commit to the realisation of the SDGs in all areas of social and economic life, as well as the activity undertaken by the competition, may soon contribute to mutual imitation of the schemes of sustainability reporting [14], and inclusion of information on the realisation of the SDGs into the company’s marketing communication [15].

Notwithstanding the above, the third of the identified patterns has the greatest number of followers. It consists in not publishing sustainability reports nor making references to either sustainability categories or directly to the SDGs in marketing communications. Low SCIMC(Pj) index values have been recorded for more than a half of the companies under analysis (eleven out of twenty). Evidently, sustainability issues are still underestimated by many business enterprises to such an extent that they fail to inform their clients on the inclusion of the same in their business strategies. It should be mentioned here that many ferry companies which do engage in sustainability-oriented activity do not post any information on the same on their websites. This is probably due to the fact that they do not see the potential such information may have in attracting prospective customers.

The second research question **(RQ2)** concerned identification of the SDGs which the ferry shipowners claim to contribute to. The analysis revealed that voyage safety (SC9) and greenhouse gas emissions (SC2) are the two categories which the ferry companies operating in the Baltic Sea Region are most committed to. Those categories are related to, respectively, SDG_3 (Good health and wellbeing), SDG_9 (Industry, Innovation and Infrastructure), SDG_11 (Sustainable cities and communities), SDG_13 (Climate action), and SDG_14 (Life below water). These SDGs are referred to most in marketing communications, but they do not constitute an exhaustive list of SDGs which ferry operators refer to, directly or indirectly.

The presence of information concerning commitment to the SDGs in selected areas only partly coincides with the weights assigned to individual SDGs by the experts. Marketing communications on the realisation of social goals definitely gives way to that on commitment to the realisation of environmental goals. While sustainability requires balanced development in all its aspects [79].

The results of the study presented herein may have a practical as well as theoretical value. In practical terms, they may help ferry operators realise that communications concerning commitment to selected SDGs may be meaningful to prospective customers and inclusion of the same in their marketing communication may bring tangible business gains. Special focus should be made on social goals, which are currently markedly neglected.

In theoretical terms, this paper fills a research gap, as the topic of marketing communications of ferry companies is practically non-existent in the literature of the subject. Moreover, the proposed SDGs Commitment Index in Marketing Communication (SCIMC) is a valuable contribution to the modern research tools, as it can be applied to assess the level of commitment to the realisation of SDGs in the conducted marketing communications both for a single enterprise and to develop rankings of enterprises.

The focus of this research are companies operating in the Baltic Sea Region. However, an extension of the research is envisioned to include other areas, with a view to obtain more universal outcomes and draw more reliable conclusions concerning current developments in marketing communications of ferry companies and inclusion of information on actions taken towards the SDGs. Application of the same research framework will make it possible to obtain comparable results and perform a reliable comparative analysis. Conclusions concerning the approach of ferry operators to building a business strategy which takes into consideration the SDGs and methods of communication of the same to customers may give an insight into relations between business activity and sustainability issues in the maritime transport and support the development of a framework of good practices in this respect.

## Supporting information

S1 Table Survey 1 results. (XLSX)

S2 Table Survey 2 results. (XLSX)
